# Enhanced Chondrogenic Capacity of Mesenchymal Stem Cells After TNFα Pre-treatment

**DOI:** 10.3389/fbioe.2020.00658

**Published:** 2020-06-30

**Authors:** Chantal Voskamp, Wendy J. L. M. Koevoet, Rodrigo A. Somoza, Arnold I. Caplan, Véronique Lefebvre, Gerjo J. V. M. van Osch, Roberto Narcisi

**Affiliations:** ^1^Department of Orthopaedics, Erasmus MC, University Medical Center, Rotterdam, Netherlands; ^2^Department of Otorhinolaryngology, Erasmus MC, University Medical Center, Rotterdam, Netherlands; ^3^Department of Biology and Skeletal Research Center, Case Western Reserve University, Cleveland, OH, United States; ^4^Division of Orthopedic Surgery, Department of Surgery, Children's Hospital of Philadelphia, Philadelphia, PA, United States

**Keywords:** mesenchymal stem cells, tumor necrosis factor-alpha, cartilage, regenerative medicine, SOXC transcription factors, chondrogenesis, tissue engineering

## Abstract

Mesenchymal stem cells (MSCs) are promising cells to treat cartilage defects due to their chondrogenic differentiation potential. However, an inflammatory environment during differentiation, such as the presence of the cytokine TNFα, inhibits chondrogenesis and limits the clinical use of MSCs. On the other hand, it has been reported that exposure to TNFα during *in vitro* expansion can increase proliferation, migration, and the osteogenic capacity of MSCs and therefore can be beneficial for tissue regeneration. This indicates that the role of TNFα on MSCs may be dependent on the differentiation stage. To improve the chondrogenic capacity of MSCs in the presence of an inflamed environment, we aimed to determine the effect of TNFα on the chondrogenic differentiation capacity of MSCs. Here, we report that TNFα exposure during MSC expansion increased the chondrogenic differentiation capacity regardless of the presence of TNFα during chondrogenesis and that this effect of TNFα during expansion was reversed upon TNFα withdrawal. Interestingly, pre-treatment with another pro-inflammatory cytokine, IL-1β, did not increase the chondrogenic capacity of MSCs indicating that the pro-chondrogenic effect is specific for TNFα. Finally, we show that TNFα pre-treatment increased the levels of SOX11 and active β-catenin suggesting that these intracellular effectors may be useful targets to improve MSC-based cartilage repair. Overall, these results suggest that TNFα pre-treatment, by modulating SOX11 levels and WNT/β-catenin signaling, could be used as a strategy to improve MSC-based cartilage repair.

## Introduction

Cartilage has a limited repair capacity and, if left untreated after damage, it will often undergo progressive, irreversible degeneration. The treatment of cartilage defects still remains challenging and novel regenerative medicine strategies are needed. Mesenchymal stem cells (MSCs) are promising cells for cell-based cartilage regeneration approaches (Caplan, 1991; Caplan and Dennis, 2006) because ease of isolation, chondrogenic potential (Johnstone et al., 1998; Pittenger et al., 1999) and anti-inflammatory properties (Kinnaird et al., 2004; Caplan and Dennis, 2006; Ren et al., 2008; van Buul et al., 2012). These properties can be affected by factors present in the microenvironment, such as pro-inflammatory cytokines. TNFα is one of the pro-inflammatory cytokines that can be present in symptomatic cartilage defects (Tsuchida et al., 2014), osteoarthritic cartilage and synovium (Chu et al., 1991; Kapoor et al., 2011; Tsuchida et al., 2014), and that contributes to the pathophysiology of osteoarthritis (reviewed by Fernandes et al., 2002; Goldring and Otero, 2011).

Exposure to TNFα during MSC chondrogenesis *in vitro* reduces the chondrogenic capacity (Wehling et al., 2009), increasing the expression of aggrecanases and decreasing expression of proteoglycans (Markway et al., 2016). However TNFα is known to be involved in several biological processes such as apoptosis, proliferation and cell survival (Brenner et al., 2015; Cheng et al., 2019). In addition, there is also evidence that TNFα can promote tissue regeneration since it can increase osteogenesis (Daniele et al., 2017) and MSC proliferation and migration (Bocker et al., 2008; Bai et al., 2017; Shioda et al., 2017). It has been shown that MSCs primed with TNFα *in vitro* survive better than control MSCs when transplanted *in vivo* (Giannoni et al., 2010). Overall these data suggest that the effect of TNFα may depend on the dynamics of exposure and that its effect may be beneficial for MSC-based tissue regeneration. Specifically, the effect on chondrogenesis of TNFα administration during MSC expansion has been incompletely investigated whether in the presence or absence of an inflamed environment during the subsequent phase of cell differentiation.

In order to increase the chondrogenic capacity of MSCs under inflammatory conditions, we hypothesized that TNFα administration during cell expansion (pre-treatment) would have a beneficial effect on the subsequent chondrogenesis performed in the presence of TNFα. Here we demonstrated that TNFα pre-treatment increases MSC chondrogenesis regardless of the presence of TNFα during differentiation and that the effect of TNFα on the chondrogenic capacity is reversible. This pro-chondrogenic effect could not be obtained by pre-treatment with interleukin 1β (IL-1β) another pro-inflammatory cytokine involved in local inflammation in the joint (Goldring and Otero, 2011). Finally, to identify a possible TNFα target pathway in the pre-treated MSCs, we investigated the levels of the SOXC protein (SOX4 and SOX11), this group of SRY-related transcription factors was previously described to be stabilized by TNFα and involved in cartilage primordia and growth plate formation (Kato et al., 2015; Bhattaram et al., 2018). In addition, we also analyzed active β-catenin levels, since SOXC can increase β-catenin protein levels (Bhattaram et al., 2014) and WNT/β-catenin signaling can increase the chondrogenic potential of MSCs (Narcisi et al., 2015).

## Materials and Methods

### MSC Isolation and Expansion

MSCs were isolated from human bone marrow aspirates from patients (17–73 years old, Table S1) undergoing total hip replacement after informed consent and with approval of the ethics committee (MEC 2015-644: Erasmus MC, Rotterdam). Patients with radiation therapy in the hip area, hematologic disorders and mental retardation or dementia were excluded from our study population. MSCs were isolated by plastic adherence and the day after seeding the non-adherent cells were washed away with PBS with 1% fetal calf serum (Gibco, selected batch 41Q2047K). They were cultured in alpha-MEM (Invitrogen), with 10% fetal calf serum, 1.5 μg/ml fungizone (Gibco), 50 μg/ml gentamicin (Invitrogen), 1 ng/ml FGF2 (AbD Serotec), and 0.1 mM ascorbic acid-2-phosphate (Sigma-Aldrich). After 10–12 days, the MSCs were trypsinized and re-seeded at a density of 2,300 cells/cm^2^. MSCs in our study were selected based on their capacity to chondrogenically differentiate, their MSC morphology (small elongated and spindle-shaped cells) and expansion capacity (cells with less than 0.15 doublings/day were excluded). To investigate whether exposure to TNFα during expansion prior to chondrogenic differentiation (pre-treatment) could inhibit the negative effect of TNFα, MSCs were pre-treated with different concentrations of TNFα (0, 1, 10, or 50 ng/ml TNFα, PeproTech) for different exposure times 24 h, 4–6 days (1 passage) or 8–10 days (2 passages), and then chondrogenically differentiated in the presence of 0 or 1 ng/ml TNFα. When indicated, MSCs were first treated with TNFα for 1 passage (4 days) followed by removal of TNFα for 1 passage (4 days) and subsequently chondrogenically differentiated in the presence of 1 ng/ml TNFα. To investigate the effect of IL-1β pre-treatment on the chondrogenic differentiation, MSCs were pre-treated for 1 passage with different concentrations of IL-1β (0, 0.1, 1, 10, and 50 ng/ml, PeproTech), followed by chondrogenic differentiation in the absence of IL-1β. MSCs from different donors are indicated as donor 1, 2, 4, 5, 6, 7, 8, or 9.

### Chondrogenic Differentiation

To obtain a 3D pellet culture, 2 x 10^5^ MSCs were centrifuged at 300 g for 8 min in polypropylene tubes. To induce chondrogenesis, the pelleted cells were cultured in DMEM-HG (Invitrogen), 1% ITS (B&D), 1.5 μg/ml fungizone (Invitrogen), 50 μg/ml gentamicin (Invitrogen), 1 mM sodium pyruvate (Invitrogen), 40 μg/ml proline (Sigma-Aldrich), 10 ng/ml TGFβ1 (R&D Systems), 0.1 mM ascorbic acid 2-phosphate (Sigma-Aldrich), and 100 nM dexamethasone (Sigma-Aldrich), referred to as chondrogenic medium (Johnstone et al., 1998). After 24 h, the medium was renewed with chondrogenic medium with or without 1 ng/ml TNFα, as indicated. Afterwards the medium was renewed two times per week for a period of 4 weeks.

### *COL2A1* Reporter Assays

Cultures of human bone marrow-derived MSCs from healthy de-identified adult volunteer donors (31–33 years old, Table S1) were established as previously described (Lennon and Caplan, 2006) after informed consent. The bone marrow was collected using a procedure reviewed and approved by the University Hospitals of Cleveland Institutional Review Board (IRB# 09-90-195). MSCs from different donors are indicated as donor 3 or 10. Lentiviral constructs for the *COL2A1* promoter were placed upstream of the Gaussia luciferase reporter gene. MSCs were transduced with the *COL2A1* reporter lentivirus as previously described for a *SOX9* reporter (Correa et al., 2018). MSCs with the *COL2A1* luciferase reporter were expanded as indicated above. At different time points during chondrogenesis, medium of MSCs with the *COL2A1* reporter was harvested 48 h after the last medium renewal. Per condition, 50 μl of the medium was transferred to a white 96-well plate and 20 μM coelenterazine substrate (NanoLight technology) was injected into the wells. The Gaussia Luciferase (Gluc) activity was measured using a GloMax-96 Microplate Luminometer (Promega) in technical duplicates.

### CD Marker Analysis

Per condition, 2 x 10^5^ MSCs were re-suspended in 500 μl FACSFlow solution (BD Biosciences) and stained with antibodies against human CD45-APC (368515, BioLegend), CD90-APC (FAB2067A, R&D Systems), CD73-PE (550257, BD Biosciences), or CD105-FITC (FAB10971F, R&D Systems), following the manufacturer's guidelines. Afterwards, the cells were fixed using 2% formaldehyde (Fluka) and were filtered through 70-μM filters. Unstained cells were used as a negative control. Samples were analyzed by flow cytometry using a BD Fortessa machine (BD Biosciences). The data were analyzed using FlowJo V10 software.

### Apoptosis Analysis

Per condition, 5 x 10^5^ MSCs were re-suspended in 1x Binding Buffer and stained with Annexin V and Propidium Iodide using manufacturer's instructions (all products from eBioscience, San Diego, USA). Samples were analyzed by flow cytometry using a BD Fortessa machine (BD Biosciences) and analyzed using FlowJo V10 software.

### (Immuno)Histochemistry

After 4 weeks of chondrogenic induction, pellets were fixed with 3.8% formaldehyde, embedded in paraffin and sectioned (6 μm). Glycosaminoglycans (GAG) were stained with 0.04% thionine solution and collagen type-2 was immunostained using a collagen type-2 primary antibody (II-II 6B3, Developmental Studies Hybridoma Bank). Antigen retrieval was performed with 0.1% Pronase (Sigma-Aldrich) in PBS for 30 min at 37°C, followed by incubation with 1% hyaluronidase (Sigma-Aldrich) in PBS for 30 min at 37°C to improve antibody penetration. The slides were pre-incubated with 10% normal goat serum (Sigma-Aldrich) in PBS with 1% bovine serum albumin (BSA; Sigma-Aldrich). Next, the slides were incubated for 1 h with collagen type-2 primary antibody, and then with a biotin-conjugated secondary antibody (HK-325-UM, Biogenex), alkaline phosphatase-conjugated streptavidin (HK-321-UK, Biogenex), and the New Fuchsine chromogen (B467, Chroma Gesellschaft). An IgG1 isotype antibody (X0931, Dako Cytomation) was used as negative control.

### DNA and Glycosaminoglycan (GAG) Quantification

After chondrogenic induction for 28 days, pellets were digested using 250 μl digestion solution containing in 1 mg/ml Proteinase K, 1 mM iodoacetamide, 10 μg/ml Pepstatin A in 50 mM Tris, 1 mM EDTA buffer (pH 7.6; all Sigma-Aldrich) for 16 h at 56°C. Next, Proteinase K was inactivated at 100°C for 10 min. To determine the DNA content, 50 μl cell lysate was treated with 100 μl heparin solution (8.3 IU/ml) and 50 μl RNase (0.05 mg/ml) solution for 30 min at 37°C. Next 50 μl ethidium bromide (25 μg/ml) was added and the samples were analyzed on a Wallac 1420 Victor2 plate reader (Perkin-Elmer) using an excitation of 340 nm and an emission of 590 nm. In case the amount of DNA was lower than 1 μg per ml, 50 μl cell lysate was treated with 50 μl heparin solution and 25 μl RNase for 30 min at 37°C. After incubation, 30 μl CYQUANT GR solution (Invitrogen) was added and samples were analyzed on a SpectraMax Gemini plate reader using an excitation of 480 nm and an emission of 520 nm. DNA sodium salt from calf thymus was used as a standard (Sigma-Aldrich). GAG content was determined using the 1,9-dimethylmethylene blue (DMB) assay, as previously described (Farndale et al., 1986). In short, 100 μl cell lysate, containing up to 5 μg GAG, was incubated with 200 μl DMB solution and analyzed using an extinction of 590 nm and 530 nm. Chondroitin sulfate sodium salt from shark cartilage was used as a standard (Sigma-Aldrich).

### mRNA Expression Analysis

After chondrogenic induction for 14 or 28 days, pellets were lysed in RNA-Bee (TEL-TEST) and manually homogenized. RNA was extracted using chloroform and purified using the RNeasy Kit (Qiagen), following manufacturer's guidelines. RNA was reverse-transcribed with a RevertAid First Strand cDNA synthesis kit (MBI Fermentas). Real-time polymerase chain reactions were performed with TaqMan Universal PCR MasterMix (Applied Biosystems) or SYBR Green MasterMix (Fermentas) using a CFX96TM PCR detection system (Bio-Rad). Primers are listed in Table S2 and the genes *GAPDH, RPS27A*, and *HPRT1* were used as housekeeping genes. The best housekeeping index (BHI) was calculated using the formula (Ct^GAPDH^^*^ Ct^*RPS*27a^^*^ Ct^*HPRT*1^)^1/3^. Relative mRNA levels were calculated using the formula 2^−ΔΔCt^.

### Adipogenic Differentiation

To induce differentiation toward the adipogenic lineage, 2 x 10^4^ cells/cm^2^ were seeded and cultured in DMEM HG (Invitrogen) with 10% fetal calf serum (Gibco), 1.5 μg/ml fungizone (Invitrogen), 50 μg/ml gentamicin (Invitrogen), 1.0 μM dexamethasone (Sigma-Aldrich), 0.2 mM indomethacin (Sigma-Aldrich), 0.01 mg/ml insulin (Sigma-Aldrich) and 0.5 mM 3-isobutyl-l-methyl-xanthine (Sigma-Aldrich) for 14 days. In order to visualize intracellular lipid accumulation, cells were fixed in 3.8% formaldehyde, treated with 0.3% Oil red O solution (Sigma-Aldrich) for 10 min and then washed with distilled water. In addition, *PPARG* mRNA expression was analyzed as indicated above.

### Osteogenic Differentiation

To induce differentiation toward the osteogenic lineage, 3 x 10^3^ cells/cm^2^ were seeded and cultured in DMEM HG (Invitrogen) with 10% fetal calf serum (Gibco), 1.5 μg/ml fungizone (Invitrogen), 50 μg/ml gentamicin (Invitrogen), 10 mM β-glycerophosphate (Sigma-Aldrich), 0.1 μM dexamethasone (Sigma-Aldrich), and 0.1 mM ascorbic acid-2-phosphate (Sigma-Aldrich) for 14–18 days. For the detection of calcium deposits (Von Kossa staining), cells were fixed in 3.8% formaldehyde, hydrated with distilled water, treated with 5% silver nitrate solution (Sigma-Aldrich) for 60 min in the presence of bright light and then washed with distilled water followed by counterstaining with 0.4% thionine (Sigma-Aldrich). In addition, *ALPL* mRNA expression was analyzed as indicated above.

### Western Blot

To test for SMAD2 activation, MSC monolayers were pre-treated for 4 days with 0 or 50 ng/ml TNFα in standard MSC growth medium, followed by serum starvation overnight (16 h) in DMEM-HG (Invitrogen) containing 1% ITS (B&D), 1.5 μg/ml fungizone (Invitrogen), 50 μg/ml gentamicin (Invitrogen), 1 mM sodium pyruvate (Invitrogen), 40 μg/ml proline (Sigma-Aldrich) and 0 or 50 ng/ml TNFα. Next, MSCs were stimulated for 30 min with 0 or 10 ng/ml TGFβ1 in the presence or absence of 1 ng/ml TNFα. To assess the SOXC proteins (SOX11 and SOX4), and (active) β-catenin levels, MSCs were pre-treated for 4 days with 0 or 50 ng/ml TNFα in standard MSC medium. Twenty-four hours prior to harvest, the medium was renewed with standard MSC growth medium containing 0 or 50 ng/ml TNFα. Western blot were made using MSC lysates prepared using M-PER lysis buffer containing 1% Halt Protease Inhibitor and 1% Halt Phosphatase Inhibitor (Thermo Scientific). Total protein concentration was determined using a BCA assay (Thermo Scientific). Eight to ten micrograms of protein was electrophoresed on a 4–12% gradient SDS-PAGE gel. Proteins were transferred to a nitrocellulose membrane (Millipore) by semi-wet transfer, followed by blocking with 5% milk powder dissolved in Tris-buffered saline containing 0.1% Tween (TBST) for 3 h. Membranes were incubated overnight at 4°C with primary antibody according to Table S3 in 5% BSA in TBST, followed by incubation at room temperature for 1.5 h with peroxidase-conjugated anti-rabbit secondary antibody (Cell Signaling, 7074S) in 5% dry milk in TBST. Proteins were detected using the SuperSignal Wester Pico Complete Rabbit IgG detection kit (ThermoFisher scientific) following manufacturer's instructions.

### Statistical Analysis

Data were analyzed using SPSS software (IBM SPSS statistics 25). Normal distribution was tested using the Kolmogorov-Smirnov test. Since the Kolmogorov-Smirnov test showed that the *COL2A1* reporter data was not normally distributed a Mann-Whitney *U*-test was applied to analyze these data. All other data were normally distributed and a linear mixed model, with the different conditions considered as fixed parameters and the donors as random parameters was applied. Bonferroni *post-hoc* tests were performed to correct for multiple comparisons. *P*-values less than 0.05 were considered statistically significant.

## Results

### MSCs Pre-treated With TNFα Had an Increased Chondrogenic Potential When Subsequently Maintained Under Stimulation by TNFα During Differentiation

**Figure 1:**
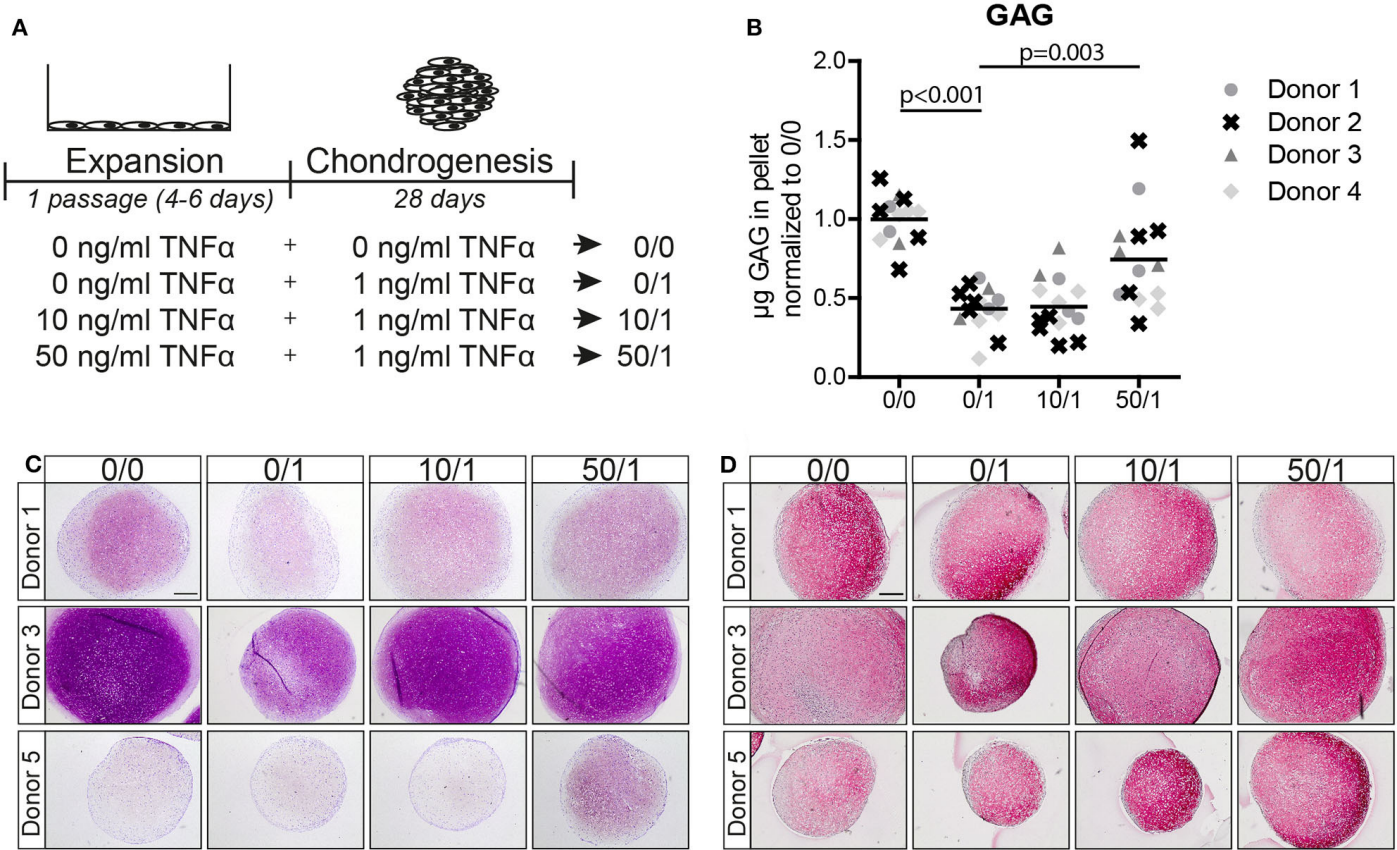
Pre-treatment of MSC monolayers with 50 ng/ml TNFα reduced the inhibitory effect of the cytokine in subsequent chondrogenic conditions. **(A)** Schematic overview of the experiment. **(B)** GAG content of MSC pellets after 28 days of chondrogenic induction. *N* = 4 donors with 2–5 pellets per donor. **(C,D)** Representative images of pellets stained for GAG with **(C)** thionine and **(D)** COL2A1 after 28 days of chondrogenic induction. *N* = 5 donors with 2–3 pellets per donor. Scale bar represents 250 μm.

To study if TNFα exposure during MSCs expansion (pre-treatment) could inhibit the negative effect of TNFα during chondrogenic differentiation, MSCs were pre-treated with different concentrations of TNFα and incubation time prior to chondrogenic differentiation in the presence of 1 ng/ml TNFα (Figure S1A). First, we confirmed that the presence of 1 ng/ml TNFα during the chondrogenic phase reduced the ability of MSCs to differentiate (Figures S1B–D; condition 0/0 vs. 0/1). Pre-treatment for 1 passage increased GAG deposition in MSC pellets after chondrogenic induction in the presence of TNFα (Figure S1C), with the larger effect occurring when the pre-treatment was performed with 10 and 50 ng/ml TNFα (Figure S1C; condition 0/1 vs. 10/1 and 50/1). Pre-treatment with TNFα for 24 h and 2 passages did not have a clear effect on chondrogenesis (Figures S1B,D; 0/1 vs. 1/1, 10/1 and 50/1). Given these observations, we performed the rest of the experiments using TNFα pre-treatment for 1 passage (Figure 1A).

Next, we analyzed MSCs from four other donors and observed that chondrogenic pellets of MSCs pre-treated for 1 passage with 50 ng/ml TNFα had a higher GAG content after 28 days of chondrogenic induction in the presence of TNFα compared to MSCs without TNFα pre-treatment (Figure 1B and Figure S2A; 0/1 vs. 50/1, *p* = 0.003), while no effect on DNA content was observed (Figure S2A). Moreover, GAG staining demonstrated an increased GAG content in the TNFα pre-treated MSCs at day 28 (Figure 1C; 0/1 vs. 50/1). Staining for collagen type-2 did not show differences (Figure 1D). No increase in GAG content was observed after pre-treatment for 1 passage with 10 ng/ml TNFα (Figures 1B,C). In order to determine whether the effect of 50 ng/ml TNFα is due to an acceleration of chondrogenic differentiation, first a non-destructive luciferase-based method was validated as a proxy for endogenous *COL2A1* expression in pellet cultures (Figure S3) and then applied to assess *COL2A1* promoter activation at day 3 and day 7 of chondrogenic differentiation. However, no significant differences were observed in *COL2A1* promoter activation at day 3 and day 7 (Figure S4A) of chondrogenesis, suggesting that TNFα pre-treatment did not increase the rate of chondrogenesis during the first week of differentiation. Subsequent analysis performed by RT-PCR on *SOX9, COL2A1*, and *ACAN* showed no significant differences at day 14 and 28 during chondrogenesis between the conditions (Figures S4A,B).

Overall, these data indicate that pre-treatment of MSC monolayers with 50 ng/ml TNFα significantly increases the chondrogenic potential of MSCs when exposed to 1 ng/ml TNFα during differentiation, but without prompting the onset of chondrocyte marker expression. For this reason, the following experiments were performed using 50 ng/ml TNFα pre-treatment.

### Pre-treatment With TNFα Increased the Chondrogenic Potential of MSCs Regardless the Presence of the Cytokine During Chondrogenic Differentiation

**Figure 2:**
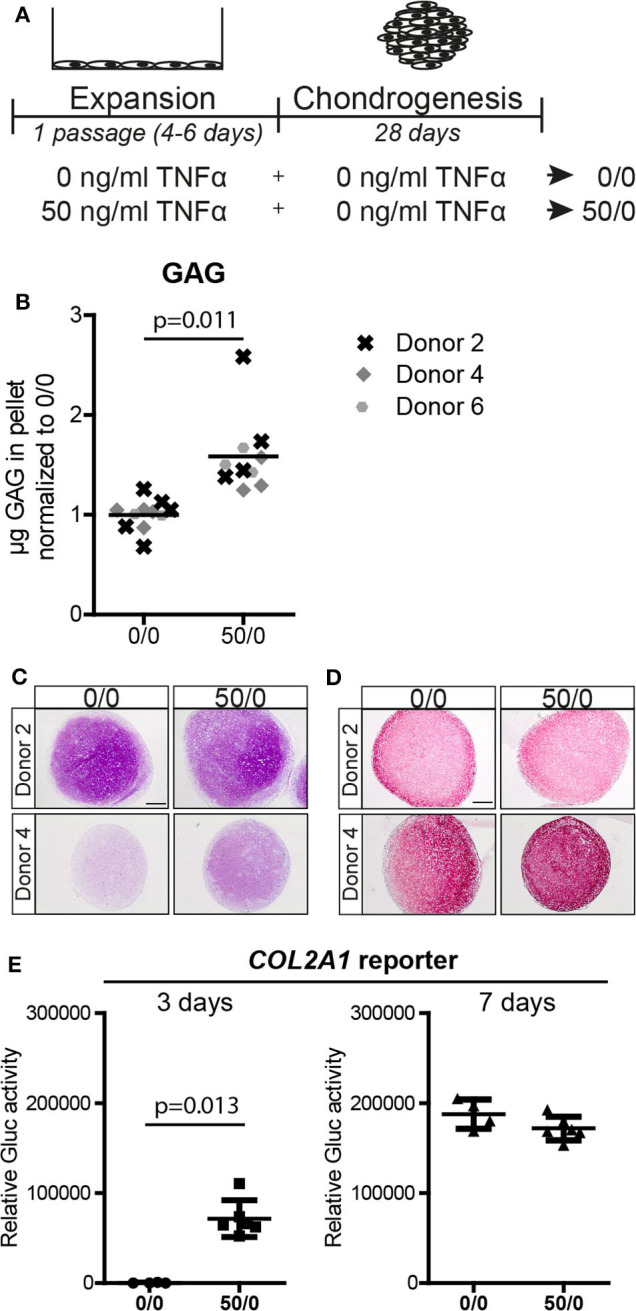
Pre-treatment with 50 ng/ml TNFα increased the chondrogenic potential**. (A)** Schematic overview of the experiment. **(B)** GAG content of MSC pellets after 28 days of chondrogenic induction. *N* = 3 donors with 2–4 pellets per donor. **(C,D)** Representative images of pellets stained for **(C)** GAG with thionine and **(D)** COL2A1 after 28 days of chondrogenic induction. *N* = 4 donors with 2–3 pellets per donor. Scale bar represents 250 μm. **(E)** Relative secreted Gaussia Lucificerase (Gluc) activity of medium from MSC pellets containing the *COL2A1* promoter reporter after 3 and 7 days of chondrogenic induction. Values represent the mean ± SD with 4–6 pellets.

Next, we tested whether the effect of TNFα pre-treatment on the chondrogenic potential of MSCs was dependent on the presence of the cytokine during the differentiation phase. MSCs were pre-treated during expansion with 0 and 50 ng/ml TNFα, followed by chondrogenic induction in the absence of TNFα (Figure 2A). Biochemical assays determined that chondrogenic pellets of MSCs pre-treated with TNFα had a higher GAG concentration (*p* = 0.011; Figure 2B and Figure S2B) and DNA content (Figure S2B). Histological staining confirmed increased GAG accumulation (Figure 2C), while no clear effect on collagen type-2 content was observed after chondrogenic induction (Figure 2D). To further investigate the effect of TNFα pre-treatment on the speed of chondrogenic induction in the absence of TNFα, we determined *COL2A1* promoter activation over time using a *COL2A1* luciferase reporter system. Analysis on 3-day pellet cultures indicated that TNFα pre-treated MSCs had enhanced luciferase activity, while no differences between the conditions were observed at day 7 (Figure 2E). These data suggest that TNFα pre-treatment accelerates chondrogenic differentiation probably via an early induction of *COL2A1* expression among other genes. These data indicate that TNFα pre-treatment increases the chondrogenic potential of the MSCs regardless of the presence of TNFα during chondrogenesis.

To better understand the effect of the TNFα pre-treatment on MSCs and the specificity for the chondrogenic lineage, we determined if TNFα increased apoptosis, expansion, and multilineage differentiation potential. No clear effect on apoptotic rates was observed after 24 h or 5 days of exposure to TNFα (Figures S5A,B), but a slight increase in MSC expansion capacity was detected after pre-treatment for 1 passage (1.4-fold difference; Figure S5C). Adipogenically induced MSCs pre-treated with TNFα showed less lipid accumulation compared to control MSCs (*p* = 0.039; Figure S5D) and a reduced *PPARG* expression, which codes for a transcription factor involved in the adipogenic differentiation process (Figure S5E). No statistically significant effect of TNFα pre-treatment on the osteogenic differentiation capacity was observed although, on average, mineralization and *ALPL* expression slightly increased (Figures S5F,G). Overall, these data suggest that TNFα pre-treatment specifically enhances the chondrogenic potential of the MSCs.

### IL-1β Pre-treatment Did Not Increase the Chondrogenic Potential of MSCs

**Figure 3:**
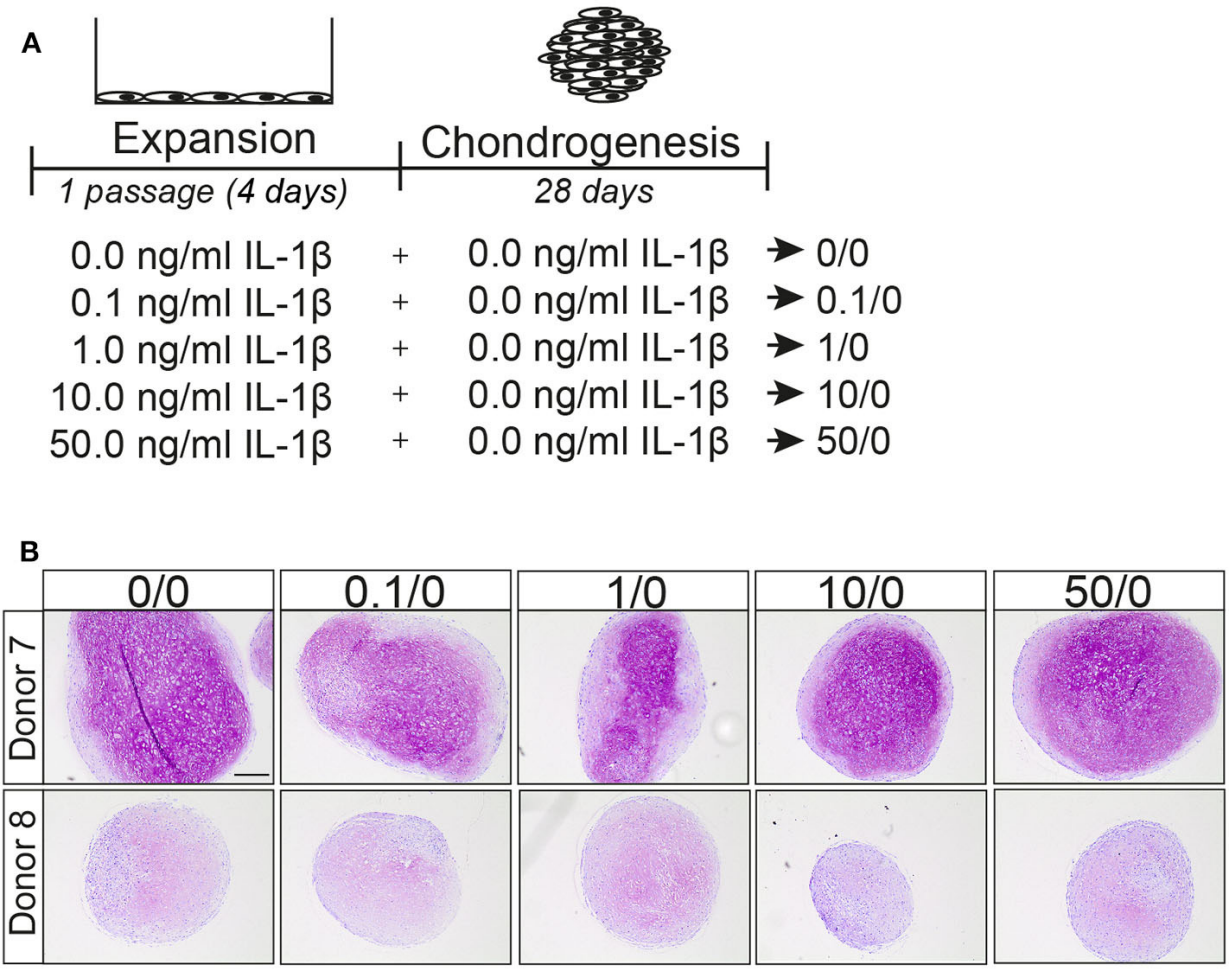
IL-1β pretreatment did not increase the chondrogenic differentiation capacity of MSCs. **(A)** Schematic overview of experiment. **(B)** GAG staining with thionine of MSCs pellets after 28 days culture in chondrogenic medium. Representative image of MSC pretreated for 1 passage with different concentrations IL-1β. *N* = 2 donors with 3 pellets per donor. Scale bar represents 250 μm.

We then investigated whether the effect of pre-treatment on the chondrogenic potential of MSCs was specific for TNFα or whether IL-1β another pro-inflammatory cytokine can have a similar effect (Figure 3A). No differences in GAG deposition were observed after pre-treatment with different concentrations of IL-1β, based on histology (Figure 3B), indicating that in contrast to TNFα, IL-1β pre-treatment for 1 passage does not increase the chondrogenic potential of the MSCs. These data suggest distinct effects of TNFα and IL-1β pretreatments on MSCs.

### The Effect of TNFα Pre-treatment on the Chondrogenic Capacity and Expression of MSC Marker CD105 Was Reversible

**Figure 4:**
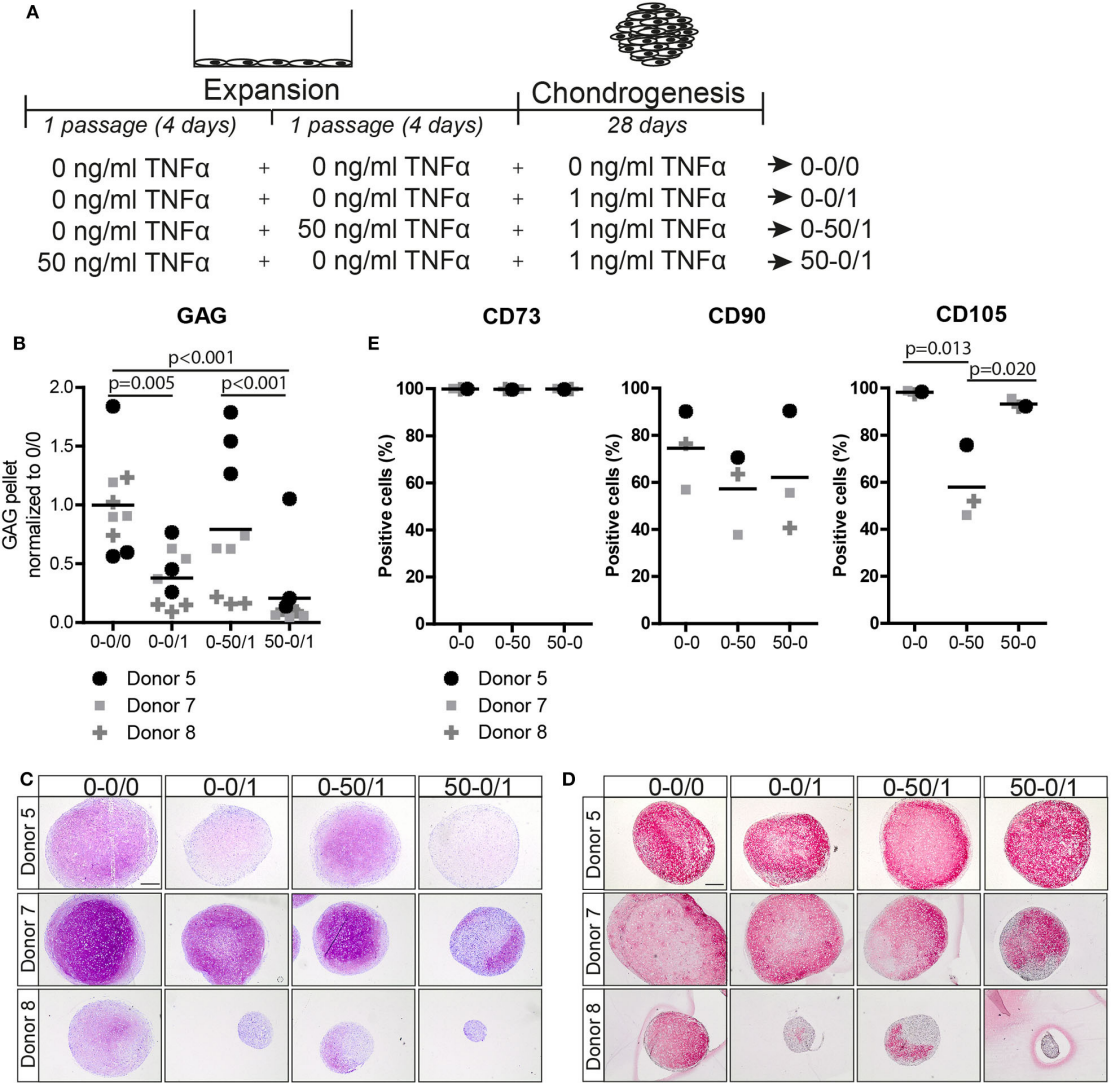
The effect of TNFα pre-treatment on chondrogenesis and MSC marker expression was reversible after TNFα withdraw**. (A)** Schematic overview of the experiment. **(B)** GAG content of MSC pellets after 28 days of chondrogenic induction. *N* = 3 donors with 3 pellets per donor. **(C,D)** Representative images of pellets stained for **(C)** GAG with thionine and **(D)** COL2A1 after 28 days of chondrogenic induction. *N* = 3 donors with 3 pellets per donors. Scale bar represents 250 μm. **(E)** Flow cytometry analysis of surface markers CD73, CD90, and CD105. The values represent the percentage of positive cells for the indicated surface marker, *N* = 3 donors.

To study whether the effect of TNFα is reversible, MSCs were pre-treated with TNFα for one passage followed by TNFα withdraw for one additional passage and subsequently subjected to chondrogenic differentiation (Figure 4A). Interestingly, GAG staining and biochemical assays showed that the positive effect of TNFα on the amount of GAG was lost after TNFα withdrawal (*p* < 0.001; Figures 4B,C and Figure S2C). No consistent effect on DNA content and collagen type-2 expression was observed after chondrogenic induction (Figure 4D and Figure S2C). To further characterize the MSCs after TNFα pre-treatment, we analyzed the expression of the MSC markers CD73, CD90, and CD105 (Dominici et al., 2006) together with the negative MSC marker CD45 (hematopoietic marker). In the control condition without TNFα pre-treatment, more than 97% of the MSCs expressed CD73 and CD105, on average 77% of the cells expressed CD90 (Figure 4E), while no cells expressed CD45 (data not shown). TNFα administration had no effect on the expression of CD73 and CD90, but it significantly decreased the number of CD105 positive MSCs (*p* = 0.013; Figure 4E), indicating that TNFα can modulate the MSC phenotype. Interestingly, the number of CD105 positive cells returned back to control levels after TNFα withdrawal for one passage (*p* = 0.020; Figure 4E). These data indicate that the effect of TNFα pre-treatment on MSC chondrogenic capacity and phenotype is reversible.

### TNFα Pre-treatment Increased SOX11 and Active β-Catenin Expression in MSCs

To elucidate how TNFα pre-treatment increases the chondrogenic differentiation capacity of MSCs we first evaluated effects on the TGFβ1 signaling pathway, since exposure to TNFα reduced the expression of the TGFβ co-receptor CD105 (Figure 4E). MSCs were then stimulated by 10 ng/ml TGFβ1 for 30 min in the presence or absence of 1 ng/ml TNFα. TGFβ1 increased pSMAD2 levels, however the levels were not altered by TNFα pre-treatment (0/1 vs. 50/1 and 0/0 vs. 50/0; Figures S6A,B). These data suggested that TNFα pre-treatment does not alter the canonical TGFβ1/SMAD2 signaling pathway in MSCs.

**Figure 5:**
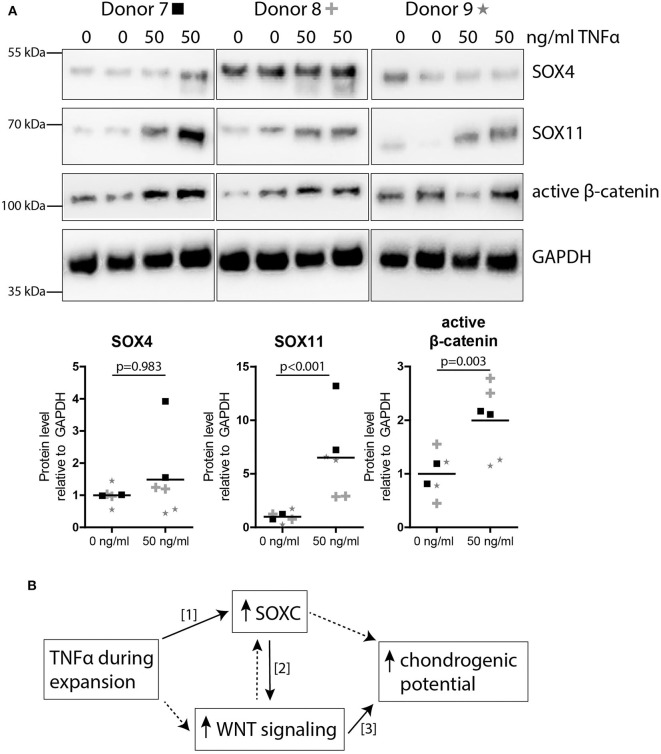
TNFα pre-treatment increased SOXC and active β-catenin expression in MSCs**. (A)** Western blot for SOXC (SOX11 and SOX4, pan-SOXC antibody) and non-phospho (active) β-catenin (Ser33/37Thr41). Below: quantification of western blot results relative to GAPDH and normalized to 0 ng/ml TNFα pre-treatment. *N* = 3 donors with biological duplicates per donor. **(B)** Possible working mechanism of TNFα pre-treatment on the chondrogenic potential of MSC. Solid lines show known interactions, [1] Bhattaram et al. *Arthritis Rheumatol* 2018. [2] Bhattaram et al. *J Cell Biol* 2014. [3] Narcisi et al. *Stem Cell Reports* 2015. Dotted lines indicate unknown interactions.

We next studied the effect of TNFα pre-treatment on SOXC proteins, SOX11 and SOX4 in MSCs. The level of SOX11 protein was significantly increased (6.5-fold; *p* < 0.001; Figure 5A), while no significant effect was observed for SOX4 (*p* = 0.983; Figure 5A). Finally, since SOXC proteins can stabilize β-catenin (Bhattaram et al., 2014), we analyzed the level of active β-catenin in the TNFα pre-treated MSCs. Interestingly, the amount of active β-catenin was increased after TNFα pre-treatment in MSCs (2.0-fold; *p* = 0.003; Figure 5A). This suggests that TNFα pre-treatment increased canonical WNT signaling in MSCs, possibly via SOXC stabilization, and thereby enhanced the chondrogenic potential (Figure 5B).

## Discussion

In this study, we demonstrated that TNFα pre-treatment of MSCs in monolayers reduced the inhibitory effect of TNFα during chondrogenic differentiation by boosting the chondrogenic capacity of these cells. This pro-differentiation effect was both temporal and specific for the chondrogenic lineage and possibly mediated by SOX11 and WNT signaling.

SOX11 is a SOXC protein which TNFα is known to stabilize in fibroblast-like synoviocytes (Bhattaram et al., 2018). SOXC genes play a crucial role in mesenchymal progenitor cell fate during skeletal development (reviewed in Lefebvre and Bhattaram, 2016). In addition, SOXC proteins are known to synergize with canonical WNT signaling via stabilization of β-catenin (Bhattaram et al., 2014). WNT signaling has been shown before to play a role in stem cell fate (ten Berge et al., 2008). We previously showed that induction of WNT signaling during monolayer increases the expansion and chondrogenic potential of MSCs (Narcisi et al., 2015, 2016). A link between SOX11 and WNT signaling has been suggested before in a study with rat MSCs where Sox11 overexpression also increased the β-catenin level and resulted in improved cartilage defect repair (Xu et al., 2019). The results of the current study suggest that SOX11 may play a role during chondrogenesis of human MSCs. Furthermore, we show that the expression of SOX11 in MSCs can be modulated by TNFα.

MSCs are a heterogeneous population of cells with known intra and inter-donor phenotypic and potency variability. This is what we also observed in our study where we used MSCs from both healthy donors and from patients undergoing total hip replacements. In addition, MSCs from patients with a broad age range were used for which we cannot exclude a possible effect of unknown underlying conditions. The differences in the chondrogenic capacity of MSCs in our study could be due to differences in cell subpopulations, since the bone marrow houses MSC subpopulations with different chondrogenic capacities (Sivasubramaniyan et al., 2018). In addition the age of the donor can have an effect on the chondrogenic capacity of MSCs (Payne et al., 2010). Although differences in chondrogenic potential were observed between MSCs from different patients, a similar effect after TNFα stimulation was detected in all cases, indicating that TNFα increases the chondrogenic potential of MSCs regardless of their chondrogenic capacity before TNFα pre-treatment.

Immunophenotyping of MSC is often used to characterize the cells (Dominici et al., 2006), even though it is a topic of discussion. We here demonstrated a clear difference in the expression of CD105, a surface marker commonly associated with the MSC phenotype (Haynesworth et al., 1992; Dominici et al., 2006), after pre-treatment with TNFα. In line with our previous work (Cleary et al., 2016), we further confirmed that CD105 is not a good marker to predict the chondrogenic potential of bone marrow-derived MSCs and, on the contrary, its expression was inversely associated with the chondrogenic capacity of MSCs. In addition, we show that the expression of CD105 can be strongly influenced by inflammatory environmental changes. This could be an explanation for contradictory published results regarding CD105 and MSCs (Majumdar et al., 2000; Kastrinaki et al., 2008; Jiang et al., 2010; Asai et al., 2014; Cleary et al., 2016). In addition, a reduced adipogenic differentiation was observed after TNFα pre-treatment. It is known that TNFα can reduce the adipogenic differentiation in 3T3-L1 pre-adipocytes by preventing *Pparg* and *Cebpa* expression (Cawthorn et al., 2007), which is in line with the reduction of *PPARG* gene expression levels that we found after TNFα pre-treatment. In agreement with other studies (Daniele et al., 2017), we observed that TNFα pre-treatment slightly increased the osteogenic differentiation capacity of MSCs. Overall, these data suggest that TNFα pre-treatment changes the immunophenotype and multipotency of MSCs.

In this study, we tested three different concentrations and incubation times and found that pre-treatment with 50 ng/ml TNFα for 1 passage (4–6 days) increased the chondrogenic capacity in a more reproducible way than the other conditions. Since a previous study indicated that 50 ng/ml TNFα can induce apoptosis in MSC (Cheng et al., 2019), we investigated apoptosis. No large effect on apoptosis was observed after addition of TNFα. Given the fact that our apoptosis rates are relatively low, we assume that the pro-chondrogenic effect of TNFα on MSCs in not due to an increased apoptotic rate. In addition TNFα can activate several transduction pathways, among which are the NF-κB, ERK, and JNK pathways (Lu et al., 2016; Bai et al., 2017). Since a 24-h pre-treatment was not sufficient to observe an effect on chondrogenesis, we assume that the effect of TNFα on the chondrogenic capacity of MSCs was not mediated via direct induction of these pathways, since they are already activated after 24 h (van Buul et al., 2012). In addition, TNFα pre-treatment for 2 passages (8–10) did not increase the chondrogenic potential suggesting that long-term exposure to TNFα during expansion does not improve the chondrogenic capacity of MSCs. Moreover, no increase in chondrogenic differentiation was observed after pre-treatment with 0.1, 1, 10, and 50 ng/ml IL-1β for 1 passage. Similar to TNFα, IL-1β is involved in joint inflammation (Goldring and Otero, 2011). This suggests that TNFα induces the pro-chondrogenic effect in MSCs via an intracellular pathway that is not activated by IL-1β. As far as we know, no previous research has investigated whether IL-1β can increase SOXC and WNT levels in human MSCs. Overall, these data indicate that not all pro-inflammatory cytokines can increase the chondrogenic potential of MSCs and that the effect seems to be specific for TNFα.

As previously reported, TNFα exposure during the chondrogenic differentiation phase reduces chondrogenesis of MSCs (Markway et al., 2016). Although the TNFα concentrations used during chondrogenic differentiation in this *in vitro* study are higher than the TNFα concentrations in post-traumatic and OA joints (4–24 pg/ml, Sward et al., 2012; Tsuchida et al., 2014; Imamura et al., 2015; Alonso et al., 2020), our data indicate that *in vitro* pre-treatment with 50 ng/ml TNFα can be beneficial for cartilage regeneration in an inflamed environment. In addition we found an association between TNFα pre-treatment and SOX11 and β-catenin activation in MSCs, therefore regulation of these pathways might improve cartilage repair in the presence of TNFα. Overall, the results of our study suggest that exposure to TNFα during the expansion phase of MSCs could improve cartilage regeneration approaches.

## Data Availability Statement

The datasets generated for this study are available on request to the corresponding author.

## Ethics Statement

The studies involving human participants were reviewed and approved by the Erasmus MC ethics committee (MEC 2015-644) and the University Hospitals of Cleveland Institutional Review Board (IRB# 09-90-195). Written informed consent to participate in this study was provided by the participants or the participants' legal guardian/next of kin.

## Author Contributions

CV: conception and design, collection of data, data analysis and interpretation, manuscript writing and final approval of manuscript. WK: collection of data, data analysis and interpretation, and final approval of manuscript. RS: data analysis and interpretation and final approval of manuscript. AC and VL: data interpretation and final approval of manuscript. GO and RN: conception and design, data analysis and interpretation, manuscript writing and final approval of manuscript. All authors contributed to the article and approved the submitted version.

## Conflict of Interest

The authors declare that the research was conducted in the absence of any commercial or financial relationships that could be construed as a potential conflict of interest.
