# Supplementary material for: Enhanced Chondrogenic Capacity of Mesenchymal Stem Cells After TNFα Pre-treatment
Source: Front Bioeng Biotechnol. 2020 Jun 30;8:658. doi: 10.3389/fbioe.2020.00658 (PMC7344141; doi:10.3389/fbioe.2020.00658)
Supplement: Supplementary file 1 [file Data_Sheet_1.docx]

***Supplementary information***

**
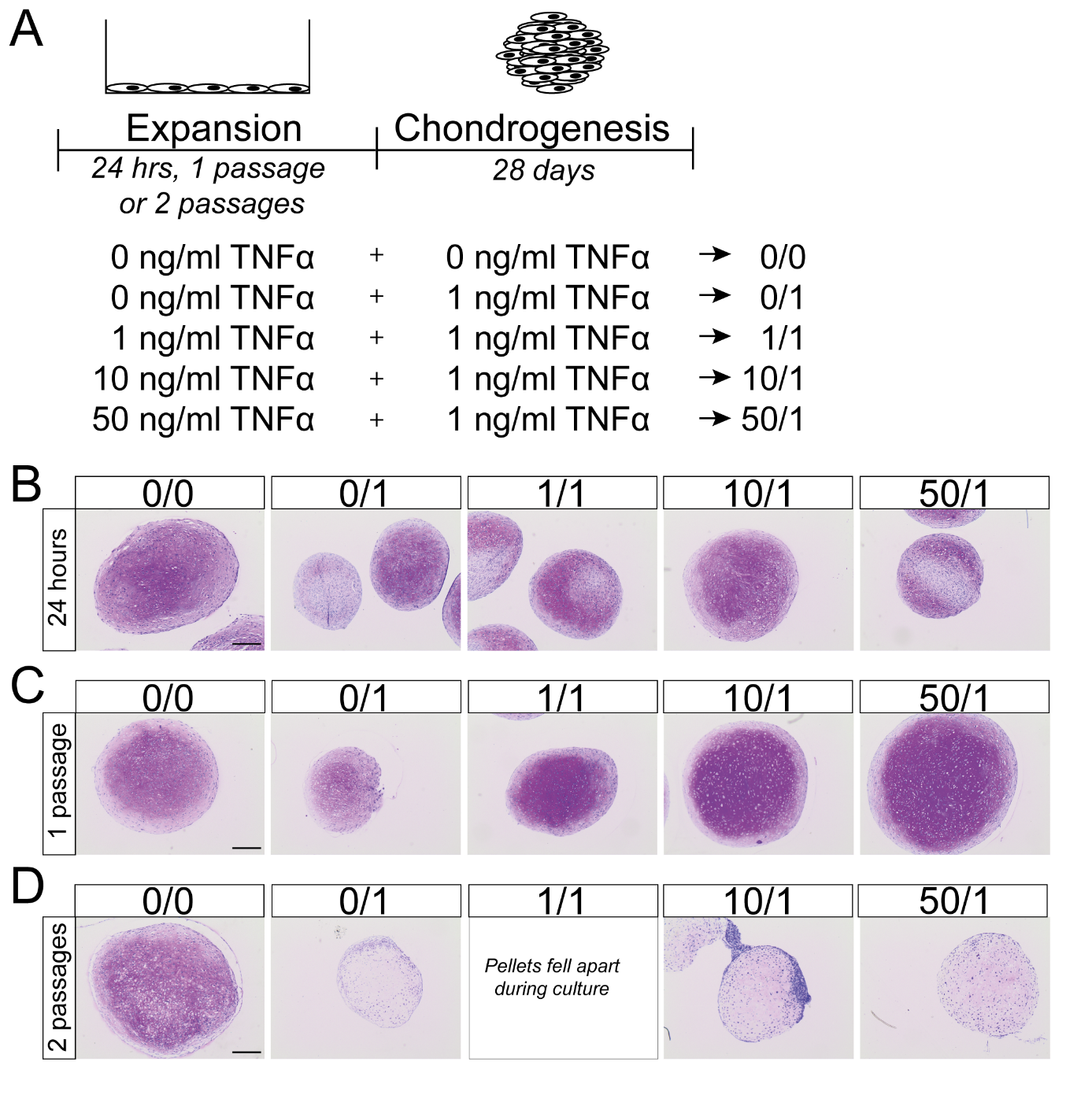
**

**Figure S1. The effect of TNFα pre-treatment of MSCs on chondrogenesis is time- and dose- dependent.** (A) Schematic overview of the experiment. (B-D) GAG staining with thionine of MSC pellets after pre-treatment in monolayer with different concentrations TNFα followed by 28 days in chondrogenic medium. Representative images of MSCs pretreated for 24 h; N=1 donor with 3 pellets per donor (B), for 1 passage (4-6 days); N=5 donors with biological triplicates (C), for 2 passages (8-10 days); N=2 donors with biological triplicates (D). Scale bar represents 250 µm.


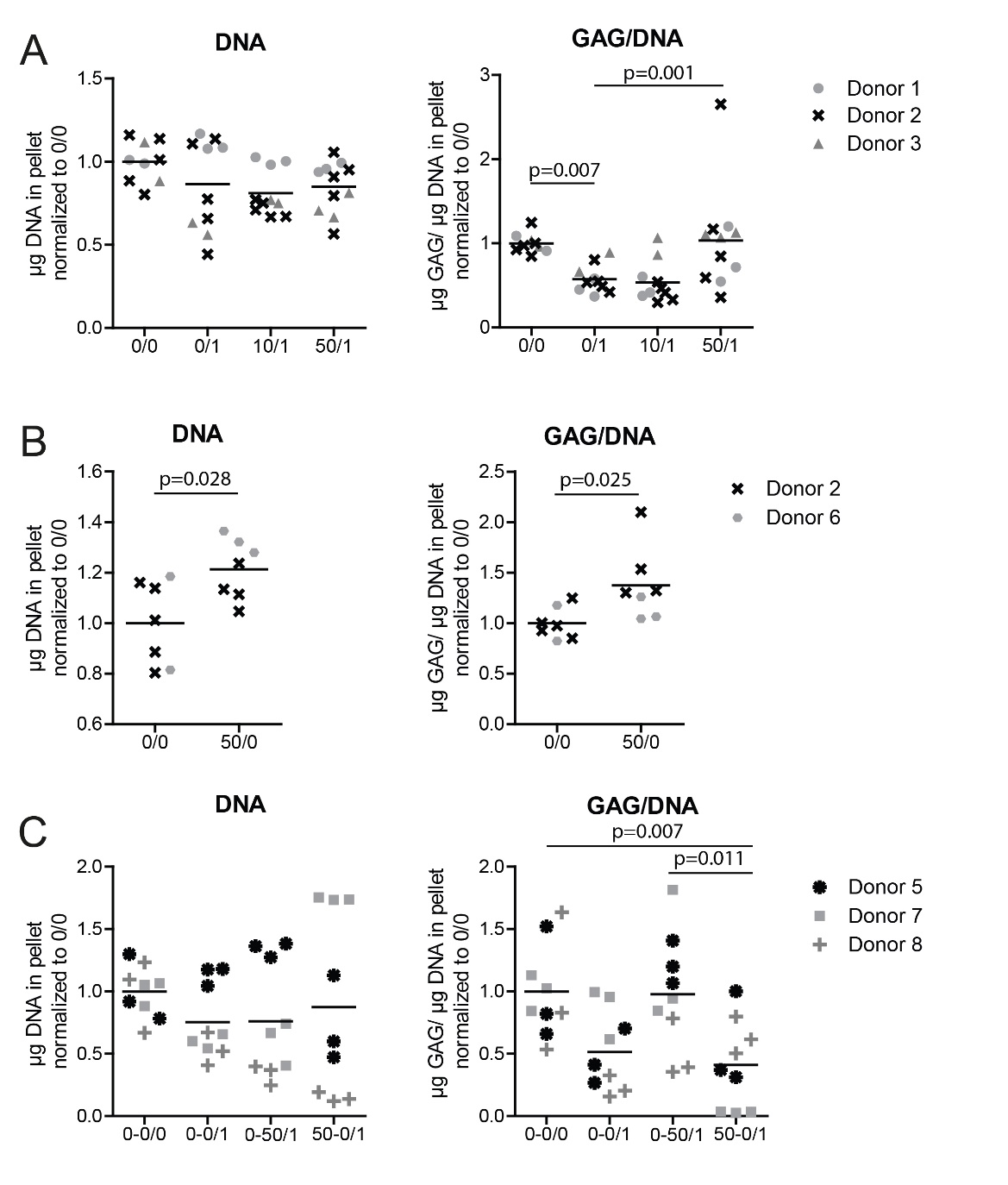


**Figure S2. DNA and GAG/DNA of MSC pellets after 28 days of culture in chondrogenic medium.** (A) Effect of TNFα pre-treatment on DNA and GAG/DNA content in cell pellets after chondrogenic differentiation in the presence of TNFα. N=3 donors with duplicates-quintuplicates per donor. (B) Effect of TNFα pre-treatment on DNA and GAG/DNA content in cell pellets after chondrogenic differentiation in the absence of TNFα. N=2 donors with biological duplicates-quintuplicates per donor. (C) Effect of TNFα withdrawal after pre-treatment on DNA and GAG/DNA content in the pellet after chondrogenic differentiation in the presence of TNFα. N=3 donors with biological triplicates per donor.


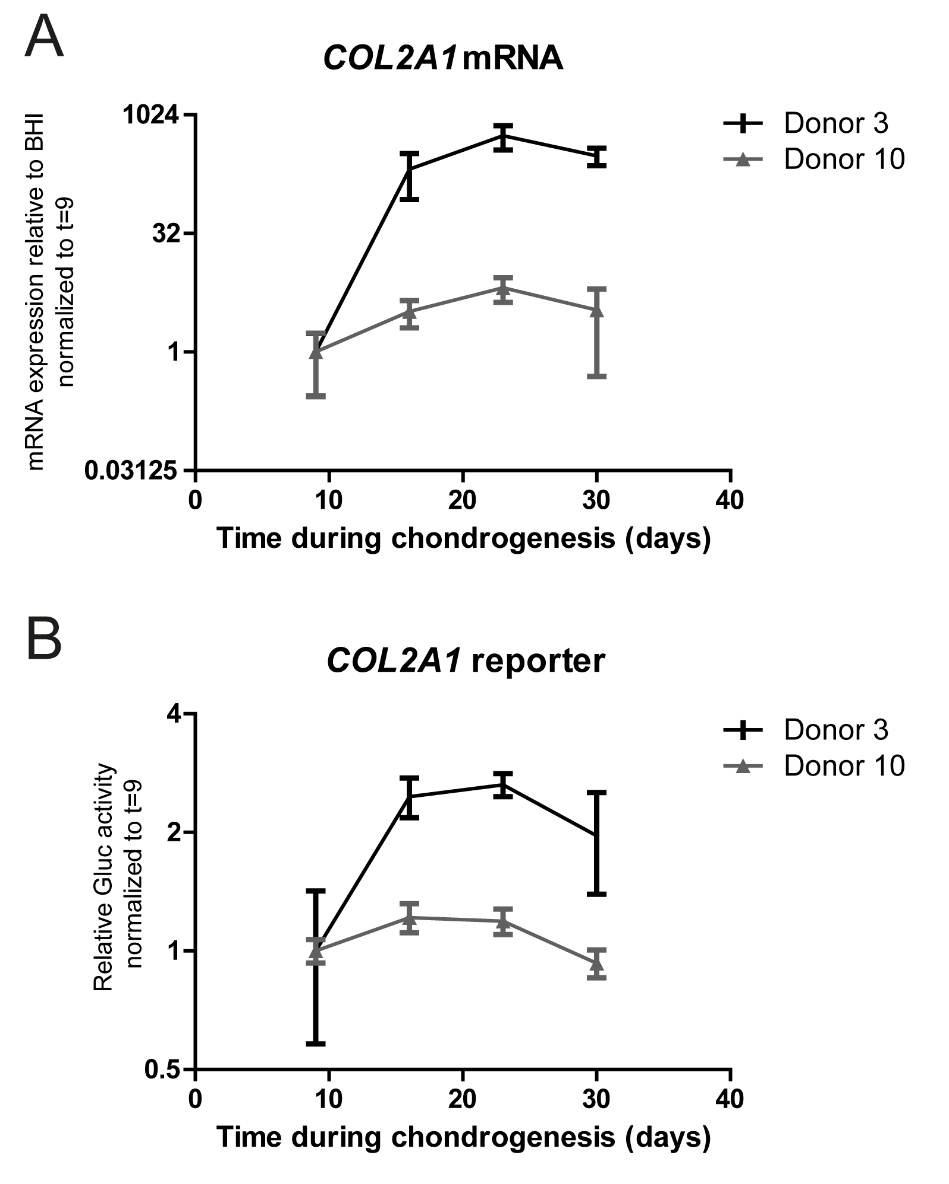


**Figure S3. Validation *COL2A1* reporter in pellets during chondrogenesis.** (A) mRNA expression relative to best housekeeper index (BHI) of pellets at t=9, t=16, t=23 and t=30 during chondrogenic differentiation. Values represent the mean ± SD, triplicates. (B) Relative Gaussia Lucificerase (Gluc) activity of medium from *COL2A1* reporter transduced pellets at various days. Values represent the mean ± SD from quintuplicates.


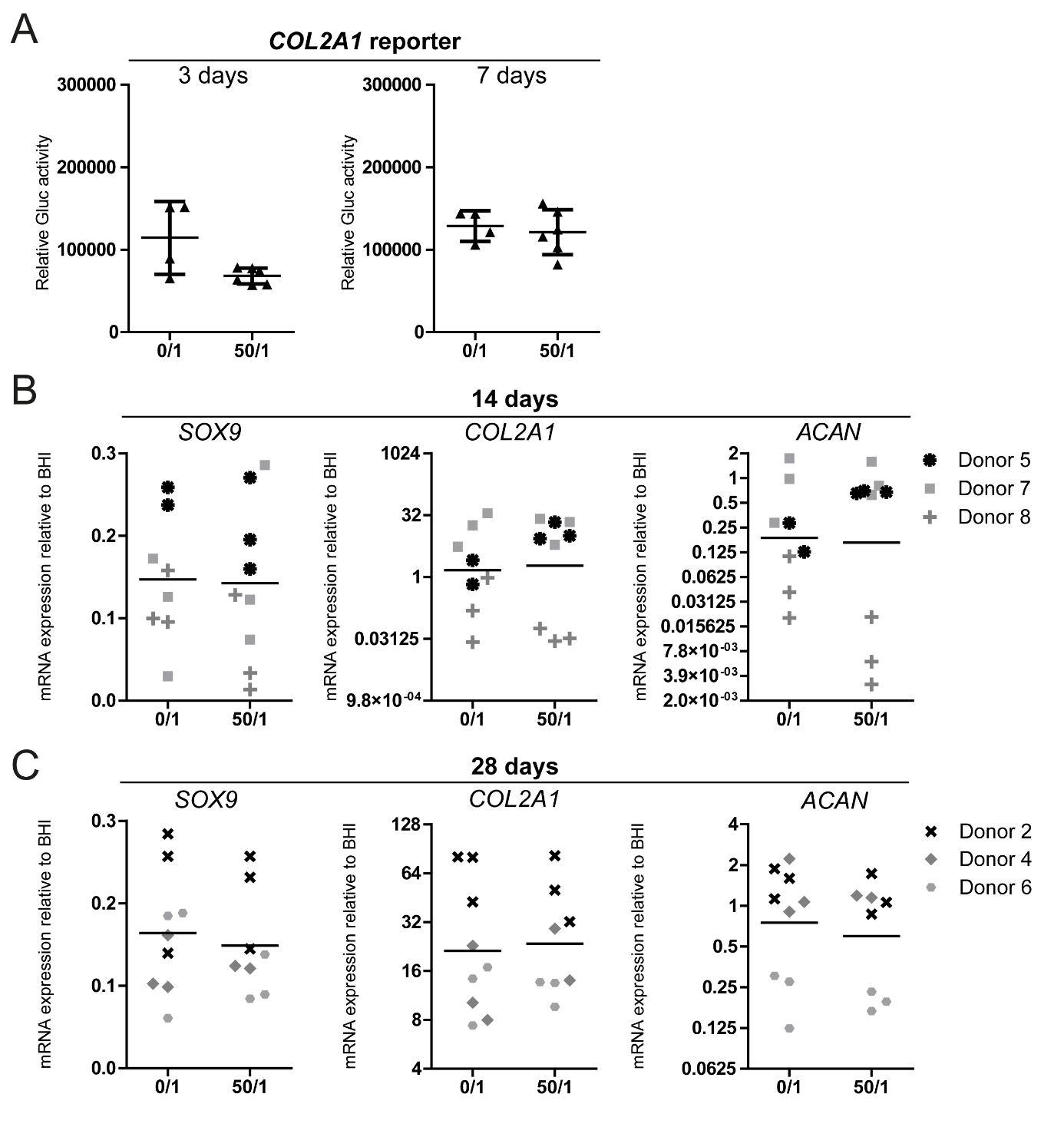


**Figure S4. 50 ng/ml pre-treatment with TNFα did not alter the expression of chondrogenic genes after chondrogenic induction in TNFα environment**. (A) Relative Gaussia Lucificerase (Gluc) activity of medium from MSC pellets containing the *COL2A1* reporter gene after 3 and 7 days of chondrogenic induction. Values represent the mean ± SD with 4-6 pellets. (B-C) *COL2A1*, *ACAN*, *SOX9* mRNA expression relative to best housekeeping index (BHI) of pellets at t=14 (B) and t=28 (C). N=3 donors with 2-3 pellets per donor.


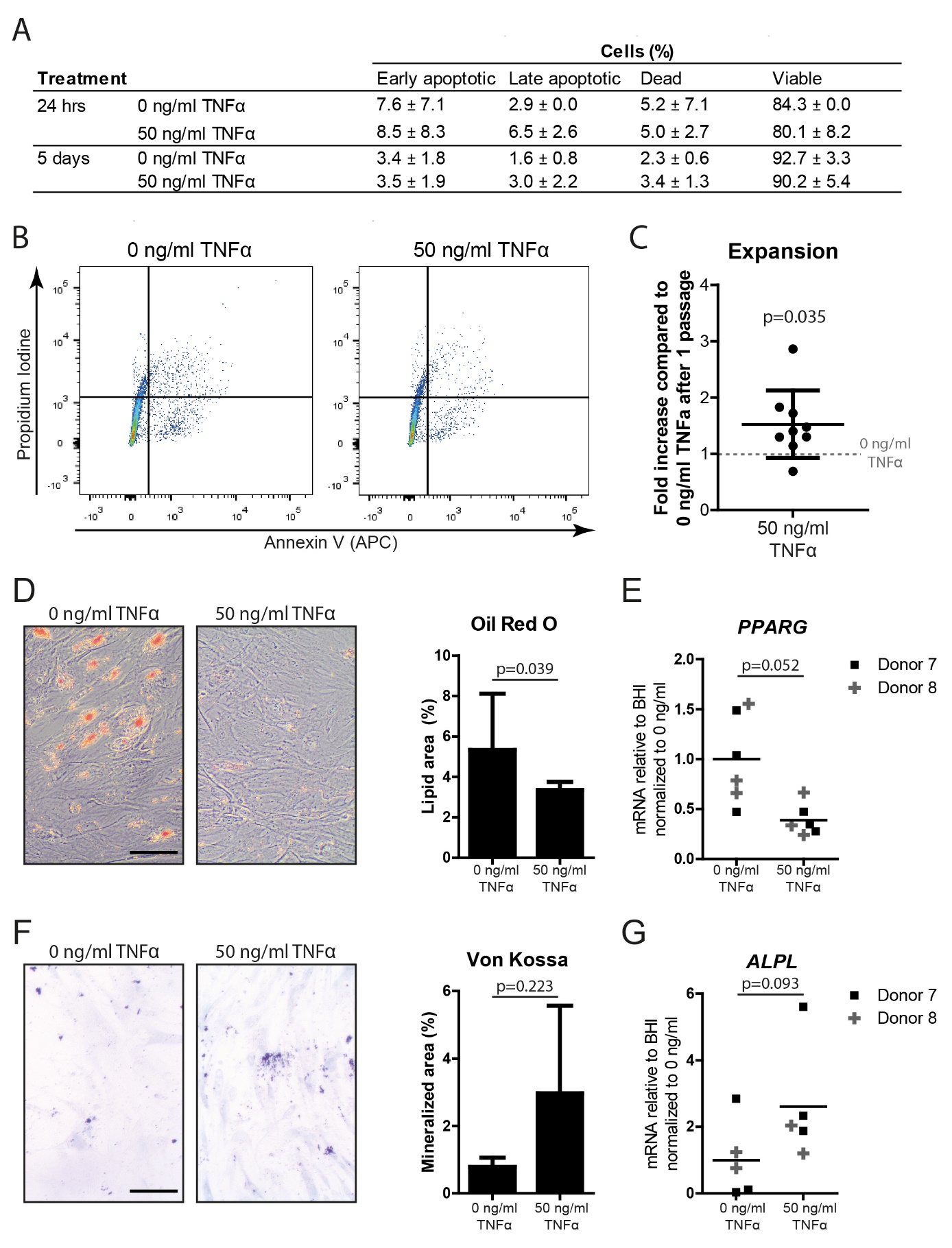
**Figure S5. TNFα pre-treatment affected the multi-potency, increased expansion and did not affect apoptosis of MSCs.** (A) Percentages of apoptotic cells determined by Annexin V and Propidium Iodide (PI) using flow cytometry. Early apoptotic cells are Annexin V/PI^+/-^, Late apoptotic cells are Annexin V/PI^+/+^, Dead cells are Annexin V/PI^-/+^, Viable cells are Annexin V/PI^-/-^. (B) Representative graph of MSCs incubated for 24 hours with 0 or 50 ng/ml TNFα followed by Annexin V and PI staining and flow cytometry analysis. (C) Cell number data during expansion after 1 passage with 0 or 50 ng/ml TNFα. N=9 donors. (D) Representative image of Oil red O staining of MSCs 21 days after adipogenic differentiation. Scale bar represents 100 µm. Right, quantification of Oil red O positive (lipids) area. N=2 donors with biological triplicates per donor. (E) *PPARG* mRNA expression of MSCs after 21 days of adipogenic differentiation. N=2 donors with three replicates per donor. (F) Representative image of Von Kossa staining of MSCs 21 days after osteogenic differentiation. Scale bar represents 100 µm. Right, quantification of Von Kossa positive (mineralized) area. N=2 donors with biological singlicate-triplicates per donor. (G) *ALPL* mRNA expression of MSCs 21 days after osteogenic differentiation. N=2 donors with 2-3 replicates per donor.


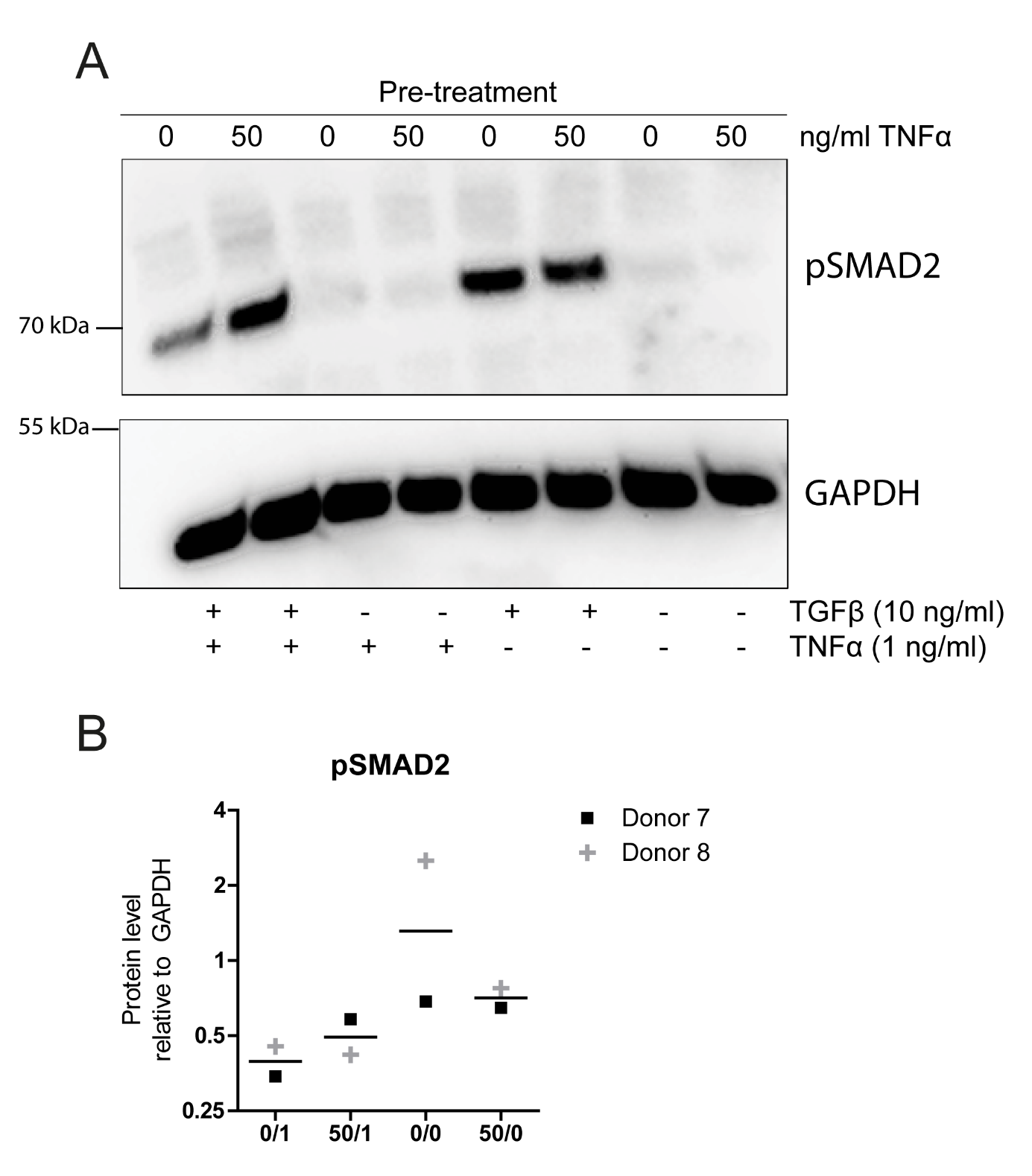


**Figure S6. TNFα pre-treatment did not alter SMAD2 activation after TGFβ1 exposure.** (A) Representative western blot showing the expression levels of phospho-SMAD2 of MSCs pre-treated for 4 days with 0 or 50 ng/ml TNFα followed by 30 min 10 ng/ml TGFβ1 and/or 1 ng/ml TNFα stimulation, N=2 donors. (B) Quantification of western blot results relative to GAPDH, N=2 donors.

**Table S1.** List of MSC donors.

| **Donor** | **Sex** | **Age (years)** | **Donor source** |
| --- | --- | --- | --- |
| 1 | F | 17 | Total hip replacement patients |
| 2 | F | 55 | Total hip replacement patients |
| 3 | M | 33 | Healthy volunteers |
| 4 | M | 42 | Total hip replacement patients |
| 5 | F | 20 | Total hip replacement patients |
| 6 | F | 73 | Total hip replacement patients |
| 7 | M | 23 | Total hip replacement patients |
| 8 | M | 50 | Total hip replacement patients |
| 9 | F | 29 | Total hip replacement patients |
| 10 | M | 31 | Healthy volunteers |

**Table S2.** List of primers used to detect mRNA levels by qRT-PCR.

| **Gene** | **Forward primer** | **Reverse primer** |  |
| --- | --- | --- | --- |
| *GAPDH* | 5’-ATGGGGAAGGTGAAGGTCG-3’ | 5’-TAAAAGCAGCCCTGGTGACC-3’ | TaqMan |
| *RPS27A* | 5’-TGGCTGTCCTGAAATATTATAAGGT-3’ | 5’-CCCCAGCACCACATTCATCA-3’ | SYBR Green |
| *HPRT1* | 5’-TTATGGACAGGACTGAACGTCTTG-3’ | 5’-GCACACAGAGGGCTACCATGTG-3’ | TaqMan |
| *COL2A1* | 5’-GGCAATAGCAGGTTCACGTACA-3’ | 5’-CGATAACAGTCTTGCCCCACTT-3’ | TaqMan |
| *ACAN* | 5’-TCGAGGACAGCGAGGCC-3’ | 5’-TCGAGGGTGTAGCGTGTAGAGA-3’ | TaqMan |
| *SOX9* | 5’-TCCACGAAGGGCCGC-3’ | 5’-CAACGCCGAGCTCAGCA-3’ | TaqMan |
| *ALPL* | 5’-GACCCTTGACCCCCACAAT-3’ | 5’-GCTCGTACTGCATGTCCCCT-3’ | TaqMan |
| *PPARG* | 5’-AGGGCGATCTTGACAGGAAA-3’ | 5’-TCTCCCATCATTAAGGAATTCATG-3’ | TaqMan |

**Table S3.** List of antibodies used to detect specific proteins in Western blots.

| **Protein** | **Antibody** | **Dilution** | **Catalog** |
| --- | --- | --- | --- |
| Pan-SOXC (Sold as SOX11), binds SOX11 more efficiently than SOX4 and SOX12 (Bhattaram, et al. 2018) | Rabbit, polyclonal | 1/1000 | Atlas antibodies, HPA000536 |
| Non-phospho (Active) β-catenin (Ser33/37/Thr41) | Rabbit, monoclonal | 1/1000 | Cell Signaling technology, 8814S |
| GAPDH | Rabbit, monoclonal | 1/1000 | Cell Signaling technology, 2118S |
| Phospho SMAD2 (Ser465/Ser467) | Rabbit, monoclonal | 1/1000 | Cell Signaling technology, 3108S |
